# Deep learning-based quantification of temporalis muscle has prognostic value in patients with glioblastoma

**DOI:** 10.1038/s41416-021-01590-9

**Published:** 2021-11-30

**Authors:** Ella Mi, Radvile Mauricaite, Lillie Pakzad-Shahabi, Jiarong Chen, Andrew Ho, Matt Williams

**Affiliations:** 1 Computational Oncology Group, Institute of Global Health Innovation, Imperial College London, London, UK; 2 Department of Radiotherapy, Charing Cross Hospital, Imperial College Healthcare NHS Trust, London, UK; 3 John Fulcher Neuro-Oncology Laboratory, Brain Tumour Research Centre, Imperial College London, London, UK; 4 Clinical Experimental Center, Jiangmen Central Hospital, Affiliated Jiangmen Hospital of Sun Yat-Sen University, Jiangmen, China; 5 Norfolk and Norwich University Hospitals NHS Foundation Trust, Norwich, UK

**Keywords:** CNS cancer, Cancer imaging, Prognostic markers

## Abstract

**Background:**

Glioblastoma is the commonest malignant brain tumour. Sarcopenia is associated with worse cancer survival, but manually quantifying muscle on imaging is time-consuming. We present a deep learning-based system for quantification of temporalis muscle, a surrogate for skeletal muscle mass, and assess its prognostic value in glioblastoma.

**Methods:**

A neural network for temporalis segmentation was trained with 366 MRI head images from 132 patients from 4 different glioblastoma data sets and used to quantify muscle cross-sectional area (CSA). Association between temporalis CSA and survival was determined in 96 glioblastoma patients from internal and external data sets.

**Results:**

The model achieved high segmentation accuracy (Dice coefficient 0.893). Median age was 55 and 58 years and 75.6 and 64.7% were males in the in-house and TCGA-GBM data sets, respectively. CSA was an independently significant predictor for survival in both the in-house and TCGA-GBM data sets (HR 0.464, 95% CI 0.218–0.988, *p* = 0.046; HR 0.466, 95% CI 0.235–0.925, *p* = 0.029, respectively).

**Conclusions:**

Temporalis CSA is a prognostic marker in patients with glioblastoma, rapidly and accurately assessable with deep learning. We are the first to show that a head/neck muscle-derived sarcopenia metric generated using deep learning is associated with oncological outcomes and one of the first to show deep learning-based muscle quantification has prognostic value in cancer.

## Introduction

Glioblastoma multiforme (GBM) is an aggressive brain malignancy with <5% 5-year survival [1]. Factors including age, performance status, tumour location, size, molecular and histological characteristics are known to be prognostic, with performance status particularly important [2]. However, performance status is subjectively evaluated, resulting in inaccuracy and high inter-observer variability, so objectively assessable indicators of frailty/physical condition such as measures of sarcopenia and skeletal muscle mass may improve prognostic assessment and treatment stratification.

Sarcopenia is associated with worse overall (OS) and progression-free survival (PFS), postoperative outcomes and chemotherapy toxicity in common cancer types [3–8]. It can be evaluated on cross-sectional imaging that is routinely performed on cancer patients. Methods with consensus include measuring cross-sectional area (CSA) of truncal abdominal musculature at L3 and psoas muscle on computed tomography (CT); these have been associated with survival in numerous cancers [4, 6, 8]. However, GBM patients routinely undergo magnetic resonance imaging (MRI) head during follow-up, rather than body CT, so there is a need for measures of sarcopenia and skeletal muscle mass derivable from MRI head. The most widely studied is temporalis muscle width (TMW), which has been identified as a skeletal muscle mass surrogate [9] and a prognostic factor for OS and PFS in GBM [10–13] and brain metastases [14, 15]. However, no studies have investigated temporalis CSA, which is likely to be a better indicator of muscle mass than width.

Currently, assessment of muscle dimensions on cancer imaging is by manual measurement or segmentation, which is time consuming, necessitates trained personnel and is prone to inter-rater inconsistency, thus limiting application to large data sets and clinical adoption. Automated muscle segmentation techniques are required for widespread use.

Automated methods have been developed for muscle segmentation, including thresholding, fuzzy *c*-means clustering, atlas/registration-based methods and shape prior modelling. One study applied range-constrained thresholding and adaptive morphological operations to segment temporalis [16], while another used Markov random field approach and region growing [17]. However, these have shortcomings: thresholding may fail when neighbouring tissues have similar intensity (as with facial muscles), atlas/registration-based methods require high computational resources and substantial time to segment each case and can fail to locate complex facial muscle structures with sufficient precision. Additionally, these methods are often semi-automatic, requiring prior knowledge, complicated feature selection and error correction, which make them challenging to build into clinical workflows.

In recent years, deep learning methods, in particular convolutional neural networks (CNNs), have achieved great success in medical image segmentation. A number of studies have demonstrated utility of CNNs in thigh [18, 19], abdominal [20–24], pelvic [25] and shoulder muscle segmentation [26, 27] for body composition analysis and sarcopenia assessment in population-based cohorts and disease conditions. However, work applying deep learning to muscle segmentation in cancer imaging or muscle segmentation on head scans is limited. In particular, no studies have provided or evaluated the use of a scalable, deep learning-based approach to quantify the temporalis muscle or its relationship to survival in brain tumour patients.

The aim of this study was to develop and validate a deep learning system for segmentation and quantification of temporalis muscle and determine whether muscle area predicts disease progression and survival in GBM.

## Methods

### Data

For training, validation and testing of segmentation performance, we used three-dimensional (3D) MRI head scans from four different data sets: an in-house glioblastoma data set (a retrospective cohort of patients with newly diagnosed GBM presenting between January 2015 and May 2018 to a tertiary medical centre) (*n* = 40), TCGA-GBM (*n* = 31), IVY-GAP (*n* = 23), and REMBRANDT (*n* = 38) (the latter three from The Cancer Imaging Archive [28]). The methods of this study have been described in part in Mauricaite et al. [29].

For the survival analysis, MRI head scans were obtained from two data sets with corresponding clinical data for patients—our in-house glioblastoma patient data set (*n* = 45) and TCGA-GBM (*n* = 51). Inclusion criteria were patients with histologically proven GBM and available baseline preoperative MRI head. The total number of glioblastoma patients in the in-house data set was 45 but we only used some of these scans as access to some was obtained after model training.

All scans were T1-weighted MRI sequences acquired with gadolinium contrast in the axial plane.

For patients in the in-house glioblastoma data set, patients’ age, sex, MGMT promoter methylation status and dates of diagnosis, death and progression were collected by LP-S who was blinded to model development and evaluation and quantification of temporalis CSA on patient scans. OS and PFS were calculated as time from date of diagnosis to death and progression, respectively. Both were censored at the date of last follow-up. For patients in the TCGA-GBM data set, clinical data were obtained from the TCGA repository. Dates of disease progression were not available.

In-house data were used in accordance with REC/HRA approval (reference: 19/LO/1763 IRAS ID: 265404) and conducted in accordance with this committee’s regulations and the Declaration of Helsinki. Public data (TCGA-GBM, IVY-GAP, REMBRANDT) was used in accordance with permissions for those data sets.

### In-house MRI scanning

All in-house scans were acquired on a 3 T Siemens scanner (Siemens Healthineers, Erlangen, Germany), with administration of intravenous gadolinium contrast. Protocols used were: two-dimensional (2D) fast low angle shot (FLASH), 3D magnetisation prepared rapid gradient echo (MPRAGE) and spin echo sequence, with field of view = 158–260 mm × 218–260 mm, matrix = 176–512 × 256–512 and slice thickness = 1–5 mm.

### Image preprocessing

MRIcroGL [30] was used to convert images from DICOM to NIfTI. Advanced Normalisation tools for Python [31] and the Intensity-Normalisation package [32] were used for bias-field correction and *Z*-score normalisation. Resampling of images to 1 × 1 voxel spacing and resizing to 256 × 256 pixels was performed.

### Image extraction

MRI head scans were sliced into 2D image sequences. For training and testing of the neural network, 366 2D axial MRI slices from 132 patient scans were extracted at levels on the craniocaudal axis between the mid-orbit and orbital roof.

### Manual segmentation

The temporalis muscle was manually segmented on the MRI slices, creating a binary mask of pixels assigned to either muscle or background class. Segmentations were performed using ITK-SNAP v3.2 [33] by consensus of two specially trained readers, RM and EM, an oncology specialty doctor, who received instruction from a senior neuro-radiologist AG with 11 years of experience. Segmentation maps were independently reviewed by MW, a senior neuro-oncologist with 9 years of experience, and JC, a neuro-oncologist with 5 years of experience. These served as reference ground truth labels in model training and evaluation. All readers were blinded to demographic and clinical characteristics of patients.

### Training, validation and test data sets

The 366 2D MR slices with corresponding ground truth labels from 132 patients were split into training, validation and test sets: 229 slices (74 patients) for training, 65 slices (27 patients) for validation, and 72 slices (31 patients) were held out for use in a test set to evaluate model performance. For patients in the validation and test sets, three slices were taken from their scan if it was from the in-house glioblastoma patient data set and two slices were taken from their scan if it was from any of the external data sets (as the in-house data set scans had significantly more total slices than scans from the external data sets). Hence, in the validation and test sets, the minimum number of slices per scan was two and the maximum was three. For patients in the training set, 3–10 slices were taken from their scan if it was from the in-house glioblastoma patient data set and 2 slices were taken from their scan if it was from any of the external data sets. More slices were taken from scans in the in-house glioblastoma patient data set as they had a larger number of total slices (some had >500 slices), in order to increase the size of the training set.

### CNN model

A deep neural network was trained for temporalis muscle segmentation, based on Ronneberger et al.’s 2D U-net architecture [34, 35], which takes MR images and ground truth muscle segmentations and yields predicted muscle segmentations. U-net is a CNN, with symmetric contracting and expanding paths. The contracting path consists of series of two 3 × 3 convolutional layers and one 2 × 2 max pooling layer, which downsample and convert input images into high-dimensional feature maps, enabling extraction of salient image features. Conversely, the expanding path consists of 2 × 2 deconvolutional and 3 × 3 convolutional layers, which upsample and retrieve image resolution from feature maps, enabling exact pixel-level localisation. The expanding path is followed by a 1 × 1 convolutional layer with sigmoid activation, which outputs the probability of each pixel being classed as muscle; this is binarised at 0.5 threshold such that each pixel is allocated to either muscle or background class. The deep neural network we constructed used 4 downsampling and upsampling operations and the same padding to convolutional kernels, reducing a 256 × 256 pixel image to a 16 × 16 data representation with 1024 channels. Regularisation was added with batch normalisation after each convolutional layer and two dropout layers (with dropout rate of 0.5) in order to reduce overfitting.

### CNN training

The U-Net was trained using stochastic gradient descent with Adam optimiser on a mini batch size of two. Optimal hyperparameters were selected based on validation loss. Initial learning rates of 0.0001, 0.0005, 0.00075, 0.001 and 0.01 were tested. Training was terminated early if the validation loss did not improve in three subsequent epochs. The best performing model was trained for 14 epochs. We implemented our model using the Tensorflow v1.14.0 [36] and Keras v2.4 [37] libraries in Python v3.6 [38]. Experiments were run on a NVIDIA GeForce RTX 2080Ti GPU.

### Loss functions

We previously compared three main categories of loss function—binary cross-entropy loss (BCEL) (distribution-based), Dice loss (DL) (region-based), and Hausdorff loss (HDL) (boundary-based)—in training and testing of the U-net model using the in-house glioblastoma data set only. BCEL and DL can be represented:

$${{Binary}}\;{{cross}}\mbox{-}{{entropy}}\;{{loss}} = - \frac{1}{N}\mathop {\sum }\limits_{i = 1}^N gi \cdot \log \left( {pi} \right) + \left( {1 - gi} \right) \cdot \log (1 - pi)$$

$${{Dice}}\;{{loss}} = 1 - \frac{{2\mathop {\sum }\nolimits_i^N pi \cdot gi}}{{\mathop {\sum }\nolimits_i^N pi + \mathop {\sum }\nolimits_i^N gi}}$$

where *N* is the total number of image pixels and *gi* and *pi* are the *i*th pixel of the ground truth and predicted segmentations, respectively. We used the DL implementation of an open-source image segmentation library [39] and HDL as derived in Ribera et al. [40, 41].

### Model evaluation

Following training, the model was evaluated on accuracy and generalisability on the test set of unseen MR images. Comparison between ground truth and predicted segmentations were made on metrics of Dice similarity coefficient (DSC), Jaccard index (JI), precision, recall and Hausdorff distance (HD) (Supplement A).

CSA of the muscle segmentation was computed using OpenCV [42]; from this, we calculated an additional evaluation metric of CSA error (Supplement B).

### Survival analysis

We analysed the association between temporalis CSA and survival in 96 patients, 45 from our in-house glioblastoma data set and 51 from the external TCGA-GBM data set. For the in-house data set, to determine CSA, a single MRI slice was taken from each of the 45 scans at the level of the superior orbital quarter, defined as equidistant between the orbital roof and mid-orbit, the level of maximal temporalis CSA. For the TCGA-GBM data set, a single MRI slice was taken from each of the 51 scans at the level of the mid-orbit.

The trained CNN model was used to generate predicted segmentations for both the left and right temporalis muscles on each MRI slice and temporalis muscle CSAs were computed from these segmentations. Average CSA for each patient was calculated as the mean of the CSAs of the two temporalis muscles.

Median temporalis CSA was determined separately for the in-house and TCGA cohort and used to dichotomise patients in each cohort to ‘low’/‘high’ CSA groups. The primary outcome was association of OS and PFS with temporalis CSA.

### Statistical methods

Summary statistics for patient characteristics and segmentation metrics are presented as mean ± SD and frequency (percentage). Paired *t* tests compared segmentation metrics obtained with different loss functions. To validate our model for the intended application of quantification of muscle as a measure of sarcopenia, similarity between CSA of manual and automated segmentations were evaluated using Pearson correlation.

We determined association of temporalis CSA and patient characteristics with Pearson correlation and independent *t* tests. Kaplan–Meier survival analysis with log rank test was used to assess differences in OS and PFS by CSA group. The association between OS/PFS and CSA was tested in univariate Cox proportional hazard models and, where significance was found, in multivariate Cox models, controlling for age and sex (MGMT methylation status was not available for sufficient patients in the in-house data set so was not used as a covariate). Confidence intervals (CIs) for all estimates of risk were given as part of sensitivity analysis. Statistical significance was set at two-tailed *p* value of <0.05. All analyses were performed using IBM SPSS v27 [43].

## Results

### Segmentation results

Table 1 summarises quantitative performance metrics obtained by comparing muscle segmentations generated by the U-net model to manually segmented reference standard for alternative loss functions. The U-net trained with DL had highest performance, outperforming BCEL and HDL. BCEL achieved comparable performance to DL, whereas performance differences between DL and HDL were significant across all metrics. Specifically, mean DSC was 0.912 for DL, significantly higher by 0.019 (*p* < 0.0005) and 0.119 (*p* < 0.0005) than BCEL and HDL, respectively. HD achieved by DL was 1.81 ± 0.39 mm, similar to 1.83 ± 0.34 mm (*p* = 0.79) for BCEL but a significant improvement over 2.18 ± 0.44 mm (*p* < 0.0005) for HDL. Hence, for subsequent model training with a larger data set, we used a U-net model trained with DL.

**Table 1:** Segmentation metrics for U-Net models trained with Dice, binary cross-entropy and Hausdorff loss functions on the in-house glioblastoma MRI data set.

Metric	Dice loss	Binary cross-entropy loss	DL vs BCEL *p* value	Hausdorff loss	DL vs HDL *p* value
DSC	0.9124 ± 0.0310	0.8931 ± 0.0397	<0.0005*	0.7938 ± 0.1326	<0.0005*
JI	0.8404 ± 0.0514	0.8091 ± 0.0623	<0.0005*	0.6730 ± 0.1421	<0.0005*
Precision	0.9253 ± 0.0557	0.9164 ± 0.0714	0.155	0.8571 ± 0.0599	<0.0005*
Recall	0.9033 ± 0.0448	0.8761 ± 0.0494	0.002*	0.7660 ± 0.1751	<0.0005*
HD (mm)	1.8129 ± 0.3895	1.8311 ± 0.3433	0.793	2.1787 ± 0.4393	<0.0005*

All figures are mean ± SD.

*DSC* Dice coefficient, *JI* Jaccard index, *HD* Hausdorff distance, *DL* Dice loss, *BCEL* binary cross-entropy loss, *HDL* Hausdorff loss.

*Significant at *p* < 0.05.

**Fig. 1:**
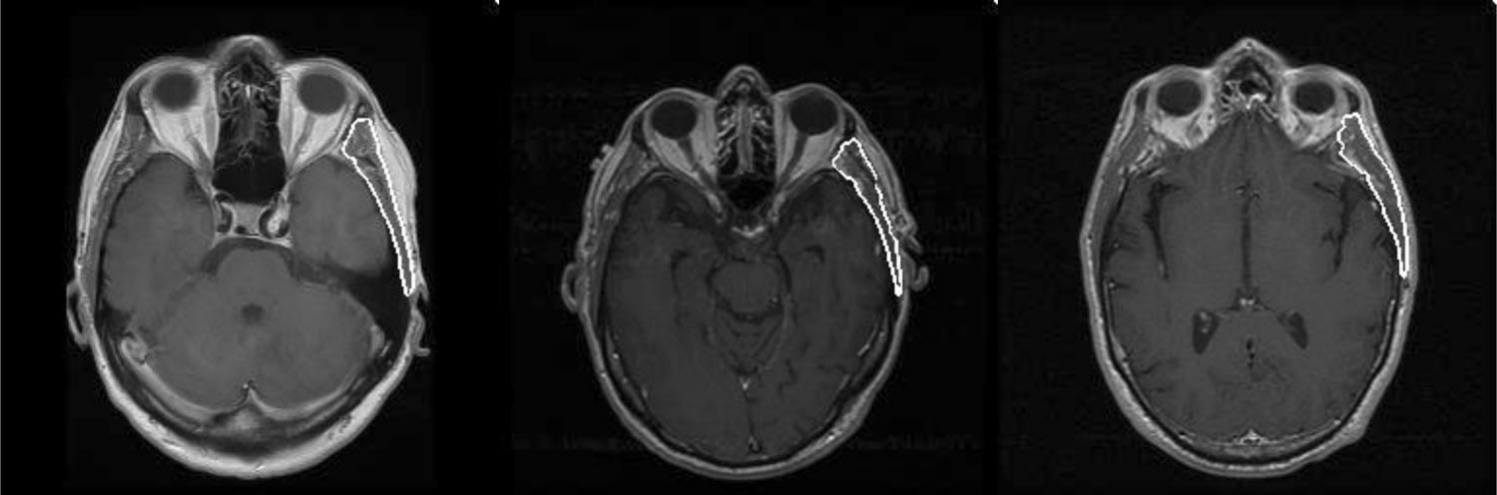
Automated temporalis segmentations. Three representative test set MRI head images (T1 weighted + GAD contrast) with overlay of predicted temporalis muscle segmentations by the neural network.

The final U-net model trained on the full data set of 366 MRI images from 132 patients segmented temporalis well. Figure 1 shows illustrative segmentation results.

Overall, the model achieved high accuracy in segmenting temporalis in the test set, with best mean DSC of 0.893 ± 0.045, JI of 0.809 ± 0.072 and HD of 1.889 ± 0.354 mm, indicating high overlap and close proximity between ground truth and automated segmentations. Mean precision and recall were similar—0.867 ± 0.077 and 0.926 ± 0.046—suggesting no bias towards over- or under-segmentation.

**Fig. 2:**
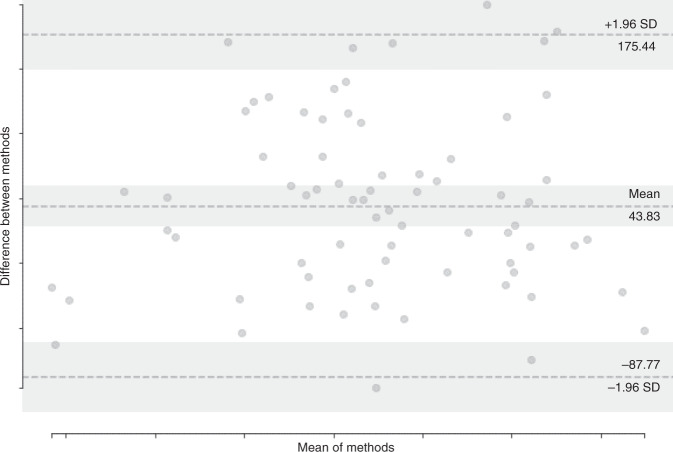
Comparison of ground truth and automated temporalis segmentation muscle areas. Bland–Altman plot comparing cross-sectional areas of manual and predicted temporalis muscle segmentations in the test set.

There was strong correlation in muscle CSA between manual and automated segmentation (*r* = 0.902, *p* < 0.0005). Average CSA error was 7.71 ± 12.17%, indicating comparable CSA measurement performance by the deep learning-based segmentation system relative to trained humans. A Bland–Altman plot (Fig. 2) shows no bias towards under- or over-segmentation.

Training on GPU took 10 min and the processing time for temporalis segmentation per novel case was 45 ms, which was substantially faster than manual segmentation by a human rater, which required 10 min per case.

### Patient characteristics

Ninety-six patients were included in the survival analysis: 45 from the in-house glioblastoma patient data set and 51 from the TCGA-GBM data set. Subjects were aged 29–78 and 23–76 years, with median (interquartile range (IQR)) age of 55 (47–63) and 58 (51–66) in the in-house and TCGA-GBM data sets, respectively. There were 34 males (75.6%) in the in-house data set and 33 males (64.7%) in the TCGA-GBM data set. MGMT promoter was methylated in 18 (40.0%) and unmethylated in 19 (42.2%) patients in the in-house data set; 8 individuals had unknown methylation status. Average follow-up was 19.2 months in the in-house data set and 15.2 months in the TCGA-GBM data set. Progression occurred in 42 (93.3%) individuals and 40 (88.9%) subjects had died at the time of analysis in the in-house data set, while 49 (96.1%) patients died in the TCGA-GBM data set. Median (IQR) OS was 18.3 (12.0–24.8) months and PFS was 8.9 (5.9–15.4) months in the in-house data set. Median OS was 14.5 (6.9–20.1) months in the TCGA-GBM data set.

### CSA and patient characteristics

**Fig. 3:**
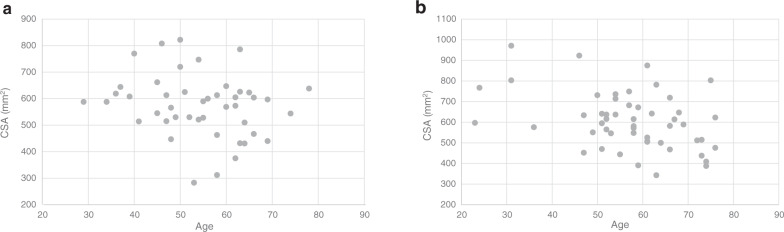
Relationship between temporalis muscle area and age. Distribution of temporalis muscle area vs age in patients in the **a** in-house glioblastoma patient data set and **b** TCGA-GBM data set. *CSA* cross-sectional area.

Mean baseline temporalis CSA was 574 ± 117 mm^2^ in the in-house glioblastoma patient data set and 605 ± 137 mm^2^ in the TCGA-GBM data set. Both were normally distributed (Kolmogorov–Smirnov test *p* > 0.2 for both). There was no significant relationship between temporalis CSA and age (*r* = −0.213, *p* = 0.16) in the in-house data set, but there was significant negative correlation between temporalis CSA and age (*r* = −0.396, *p* = 0.004) in the TGCA-GBM data set; Fig. 3 shows the distributions of CSA with age in the in-house and TCGA-GBM data sets. Males had significantly higher CSA than females in both the in-house (607 ± 100 vs 472 ± 109 mm^2^, *p* < 0.0005) and TCGA-GBM data sets (667 ± 113 vs 492 ± 101 mm^2^, *p* < 0.0005).

### CSA and survival

**Fig. 4:**
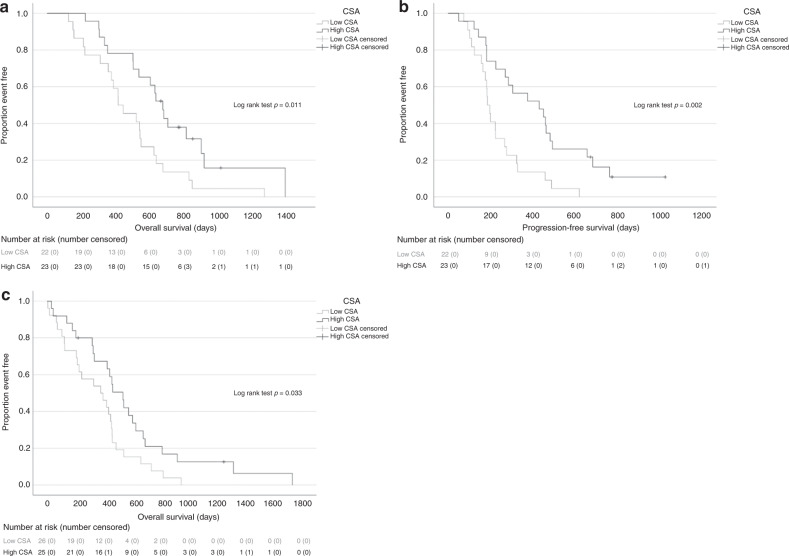
Relationship between temporalis muscle area and survival in glioblastoma. Kaplan–Meier survival curves for overall survival (**a**) and progression-free survival (**b**) by temporalis muscle area group in the in-house glioblastoma patient data set and overall survival by temporalis muscle area group in the TCGA-GBM data set (**c**). *CSA* cross-sectional area.

In the in-house glioblastoma patient data set, median baseline temporalis CSA was 588 mm^2^, which was used to dichotomise individuals to ‘low’ or ‘high’ CSA groups (*n* = 22 and *n* = 23, respectively). OS and PFS was significantly longer in patients with high CSA (median OS 22.4 (16.9–27.4) vs 14.5 (9.7–21.1) months, *p* = 0.011; median PFS 14.3 (6.1–21.9) vs 6.4 (5.0–9.6) months, *p* = 0.002) (Fig. 4a, b). In multivariate Cox models, adjusting for age and sex, CSA was an independently significant predictor for OS (hazard ratio (HR) 0.464, 95% CI 0.218–0.988; *p* = 0.046) and PFS (HR 0.433, 95% CI 0.218–0.860; *p* = 0.017) (Table 2). In the TCGA-GBM data set, median baseline temporalis CSA was 595 mm^2^, with 26 and 25 patients in the ‘low’ and ‘high’ CSA groups, respectively. OS was significant longer in patients with high CSA (15.4 (8.9–22.8) vs 12.9 (4.1–15.6) months, *p* = 0.033) (Fig. 4c). Multivariate Cox models for death for high CSA (vs low) yielded HR of 0.466 (95% CI 0.235–0.925; *p* = 0.029) so CSA was independently significant for OS (Table 2). Age was also an independent prognostic factor in both data sets.

**Table 2:** Hazard ratios for overall and progression-free survival by temporalis muscle area group for in-house glioblastoma patient and TCGA-GBM data sets.

Patients	Univariate		Multivariate	
	HR (95% CI)	*p* value	HR (95% CI)	*p* value
In-house glioblastoma patient data set—OS				
CSA	0.444 (0.233–0.845)	0.013*	0.464 (0.218–0.988)	0.046*
Age	NA	NA	1.036 (1.002–1.070)	0.036*
Sex	NA	NA	1.430 (0.622–3.287)	0.399
In-house glioblastoma patient data set—PFS				
CSA	0.367 (0.190–0.707)	0.003*	0.433 (0.218–0.860)	0.017*
Age	NA	NA	1.028 (0.998–1.059)	0.067
Sex	NA	NA	0.938 (0.436–2.014)	0.869
TCGA-GBM data set—OS				
CSA	0.536 (0.299–0.961)	0.036*	0.466 (0.235–0.925)	0.029*
Age	NA	NA	1.029 (1.004–1.056)	0.025*
Sex	NA	NA	1.887 (0.918–3.880)	0.084

All figures are HR (95% CI).

*OS* overall survival, *PFS* progression-free survival, *HR* hazard ratio, *CSA* cross-sectional area, *NA* not applicable.

*Significant at *p* < 0.05.

## Discussion

We have developed and validated a novel deep learning-based system for rapid and accurate temporalis muscle segmentation and quantification, with performance equivalent to but much quicker than trained humans. We show that temporalis muscle area is an independently significant prognostic marker for survival in GBM, corroborating previous evidence on TMW. To the best of our knowledge, this is the first study to demonstrate an association between a head/neck muscle-derived sarcopenia metric and clinical outcomes in cancer using deep learning. This is also one of the few studies, thus far, to combine both muscle segmentation and quantification using deep learning and show that such an automatically generated sarcopenia metric is significantly associated with oncological outcomes, supporting the possibility of a scalable approach to sarcopenia assessment on routine imaging in cancer.

Our technique produced generally better segmentation than previous approaches for the same muscle: previous models achieved DSC of 0.86 for masticatory muscles [44], 0.826 and 0.788 for masseter and temporalis [17] and 0.902 for temporalis [16], compared to DSC of 0.893 in this study. Our DSC is also comparable to previous models for segmentation of other muscles, for example, abdominal (DSC 0.90–0.97) [20–24], thigh (DSC 0.90–0.97) [18, 19] and shoulder (DSC 0.71–0.88) [26, 27]; our precision and recall of 0.867 and 0.926 is comparable to 0.93 and 0.91 for abdominal muscle [20]; and our HD of 1.889 mm is better than an existing model for masticatory muscles (8.2 mm) [44] as well as those for thigh (2.3–8.2 mm) [18] and lumbar abdominal (4.6–7.9 mm) muscles [22]. Thus, our model performed well, notwithstanding known challenges with facial muscles, e.g. homogeneous intensity to surrounding structures, shape complexity and significant anatomic variability. We found U-net trained using DL performed best, in keeping with DL being identified as superior for pelvic floor muscle segmentation [25].

There was a significant negative correlation between temporalis CSA and age in the TCGA-GBM data set but not in the in-house glioblastoma patient data set (although the trend was present so this difference between the two data sets may not be clinically significant). The former finding of a significant relationship is in line with general consensus of a negative correlation between muscle mass and age [45]. The latter finding of no significant relationship is similar to previous TMW studies [10–12, 14, 15]. It could be explained by disparity between chronological and biological age, the latter being more defined by frailty and physical condition. Thus, temporalis CSA may provide information not captured by age. We identified males to have significantly higher temporalis CSA than females, consistent with a study of lumbar abdominal muscle CSA [22], lending support to its use as a surrogate for skeletal muscle mass.

We found higher temporalis CSA was associated with significantly longer survival and time to progression in GBM with HR of 0.464 and 0.466, and HR of 0.433, consistent with studies of TMW reporting HRs of 0.41–0.79 for OS and 0.46–0.77 for PFS [10–13]. Sarcopenia is a key feature of cancer-related cachexia. The mechanism linking lower temporalis CSA to worse survival outcomes likely reflects physical inactivity, nutritional deficiency and glioblastoma-related catabolic, paraneoplastic and inflammatory processes. Additionally, sarcopenic patients may tolerate surgery/chemoradiotherapy poorly, leading to toxicity or early discontinuation of therapy, and thus accelerated progression and death. Our results are in line with recently published studies this year, which show that muscle CSA at the L3 vertebral level on CT imaging, as assessed by a CNN, was significantly associated with survival in advanced cancer, and that greater muscle loss, as assessed by an AI-based volumetric technique, was a poor prognostic factor for OS [46, 47].

### Implications for clinical practice

Our deep learning-based muscle segmentation and quantification tool has potential utility in bettering prognostic estimates in GBM and personalised treatment decisions, e.g. stratification to shorter, hypo-fractionated radiotherapy or temozolomide monotherapy, for which there is evidence of better outcomes in frail patients [48–50]. Our work suggests the possibility of using deep learning-based screening of sarcopenia in cancer care, without additional scanning time, cost or radiation exposure; this could inform muscle preservation interventions, such as nutrition, physiotherapy and pharmacotherapy [51–53]. Our tool is time and memory efficient, is applicable to large data sets and real-time assessment without specialist hardware and thus can feasibly be deployed in a routine clinical workflow.

### Study limitations

This is a retrospective study and we had a limited data set. However, our training, validation and test data sets included MRI scans from four different data sets, three of which are external, and MR images at multiple orbital levels, heterogeneous in field of view and pixel resolution, acquired with machines from different manufacturers using different protocols; our model’s robust performance indicates generalisability. In the survival analysis, we included patients from both our own institution and an external validation data set. However, given the limited number of patients, one can interpret the survival analysis as exploratory. The segmentation system was trained for 2D rather than 3D segmentation, common in muscle quantification due to the difficulty of creating good quality manual 3D segmentations, making 3D models more prone to error and reliant on post-processing manual correction. 3D model training and application is also substantially slower with greatly increased computational memory cost, and it is unclear whether 3D temporalis segmentation would be of additional prognostic value. To ameliorate any potential loss of information with 2D segmentation (compared to 3D), we explored the relationship between temporalis muscle area at different orbital levels with survival, similar to previous studies that used the same anatomical landmarks [12]; the existence of a significant relationship at multiple orbital levels indicates generalisability. Our reason for exploring the robustness of the relationship at different orbital levels was also to facilitate our further work to develop a fully automated pipeline for temporalis segmentation with automatic slice selection based on orbital landmarks. However, we recognise that using different landmarks in the two data sets in survival analysis introduces a degree of inconsistency in our current methods and means we cannot directly combine the results of the two data sets; a future pipeline will automatically select slices based on a consistent landmark.

## Conclusion

Our findings highlight temporalis muscle area as a non-invasive digital prognostic biomarker that can be automatically, rapidly and accurately assessed using deep learning, with feasible integration into routine clinical care. Our tool in its current form is semi-automated as it requires manual slice selection; however, we are currently developing a fully automated pipeline for temporalis segmentation including automatic slice selection using orbital landmarks. Future work will also involve prospective studies on larger cohorts.

## Supplementary information

Supplementary Material

## Data Availability

The in-house data sets used and/or analysed during the current study are available from the corresponding author on reasonable request. The TCGA-GBM, IVY-GAP and REMBRANDT external data sets used and/or analysed during the current study are available from The Cancer Imaging Archive repository (https://wiki.cancerimagingarchive.net/display/Public/).

## Code availability

Code used in the study is available at our GitLab repository https://gitlab.com/computational.oncology/temporalissegmentation and from the corresponding author on reasonable request.
