# Supplementary material for: Deep learning-based quantification of temporalis muscle has prognostic value in patients with glioblastoma
Source: Br J Cancer. 2021 Nov 30;126(2):196–203. doi: 10.1038/s41416-021-01590-9 (PMC8770629; doi:10.1038/s41416-021-01590-9)
Supplement: Supplementary file 1 — Supplementary Material [file 41416_2021_1590_MOESM1_ESM.docx]

# Supplementary Material

**Supplement A - Segmentation metrics**

Dice coefficient and Jaccard index are measures of overlap between manual and automated segmentations. Precision and recall are measures of over- and under-segmentation. These were calculated as follows:

$$Dice similarity coefficient \left( DSC \right)= \frac{2 |gt\cap pr|}{\left| gt \right|+|pr|}$$

$$Jaccard index \left( JI \right)= \frac{|gt\cap pr|}{|gt\cup pr|}$$

$$Precision= \frac{TP}{TP+FP}= \frac{|gt\cap pr|}{|pr|}$$

$$Recall= \frac{TP}{TP+FN}= \frac{|gt\cap pr|}{|gt|}$$

where *gt* and *pr* are the ground truth and predicted segmentations. Values range between [0,1] with 1 being best. We used open-source implementations of precision and recall (<https://scikit-learn.org/stable/>).

Hausdorff distance is a measure of spatial distance between manual and automated segmentations. Hausdorff distance is the maximum distance between a point on the boundary of one segmentation and the closest point on the boundary of the other segmentation and is an indicator of largest segmentation error. It is calculated as follows:

$$Hausdorff distance \left( HD \right)= max\{\max_{gi \in gt} \min_{pi \in pr} dist\left( gi, pi \right),\max_{pi \in pr} \min_{gi \in gt} dist\left( pi, gi \right)\}$$

where *gt* and *pr* are ground truth and predicted segmentations, *gi* and *pi* are points on *gt* and *pr* and *dist* is the Euclidean distance. It takes values from 0mm upwards with 0mm being most ideal. We used an open-source implementation of Hausdorff distance (<https://www.scipy.org/>).

**Supplement B - Calculation of muscle cross-sectional area and cross-sectional area error**

Calculation of CSA used the following steps:

- Determination of number of muscle pixels in segmentation image using OpenCV
- Calculation of area fraction of muscle in segmentation image by dividing number of muscle pixels by pixel resolution of segmentation image (256 x 256 pixels = 65536 pixels)
- Calculation of number of muscle pixels in input MR image by multiplying area fraction with pixel resolution of input MR image
- Calculation of area per pixel of input MR image by dividing image dimensions by pixel resolution of input MR image
- Calculation of area of muscle by multiplying number of muscle pixels with area per pixel of input MR image

CSA error is the standardised absolute percentage area difference between the ground truth and automated segmentations. It is calculated as follows:

$$CSA error= \frac{|gt-pr|}{|gt|} x 100$$
